# Supplementary material for: Perceptions, Knowledge, and Practices Concerning Indoor Environmental Pollution of Parents or Future Parents
Source: Int J Environ Res Public Health. 2020 Oct 21;17(20):7669. doi: 10.3390/ijerph17207669 (PMC7589174; doi:10.3390/ijerph17207669)
Supplement: Supplementary file 1 [file ijerph-17-07669-s001.pdf]

International Journal of
Environmental Research and Public Health

MDPI

Supplemental file :

Document S1 : Questions exploring practices.

Air Renewal and combustion :

1) Do you allow people to smoke inside your home? (yes/no)
   If so, how often?
- rarely or never
- from time to time (less than once a week)
- several times a week (one to 3 times a week)
- 4 or more times a week

2) In winter, how often is your dwelling ventilated with windows open for more than 10 minutes?
- rarely or never
- from time to time (less than once a week)
- several times a week (one to 3 times a week)
- 4 or more times a week

3) In the summer, how often is your home ventilated with windows open for more than 10 minutes?
- rarely or never
- from time to time (less than once a week)
- several times a week (one to 3 times a week)
- 4 or more times a week

4) How often do you use scented candles in your home?
- rarely or never
- from time to time (less than once a week)
- several times a week (one to 3 times a week)
- 4 or more times a week

5) How often do you use incense in your home?
- rarely or never
- from time to time (less than once a week)
- several times a week (one to 3 times a week)
- 4 or more times a week

6) How often do you (yourself) use a deodorant spray in your toilet?
- rarely or never
- from time to time (less than once a week)
- several times a week (one to 3 times a week)
- 4 or more times a week

7) How is your home ventilated?
- Functional controlled mechanical ventilation (CMV)
- ducts, natural ventilation grids
- no device or does not work
- I don't know

8) Is the CMV in your home cleaned at least once a year? (yes/ no)

9) At what temperature do you heat your home on average? ________________

10) Do you have your heating installations checked by a professional regularly (at least once a year)? (yes/ no)

11) Do you have a fireplace or stove? (yes/ no)
 If so, do you have them swept regularly (at least once a year)? (yes/ no)

Hygiene and cosmetics:

12) For your hygiene (body, hair and teeth), do you check the composition of the products you use? (yes/ no)
 If so, why? ____

13) Do you use a mobile application to choose these hygiene products? (yes/ no)

14) For your esthetics, among the cosmetics below, check those you use regularly (every day or almost every day, all year round)?
- foundation deodorant
- moisturizing face cream powder
- moisturizing body cream perfume
- moisturizing hand cream shaving foam
- aftershave lip balm
- lipstick mascara
- eye pencil gel or lacquer
- other(s) (specify) : ____

15) Among the following products, check the ones you use and indicate their frequency:
- hair dyes: ____
- nail polish : ____
- face mask: ____
- hair mask : ____

Textile:

16) Do you have your clothes cleaned regularly by a dry cleaner? (yes/ no)
 If so, how often? ____

17) Do you wash your textile before the first use?
- never or rarely
- occasionally
- regularly

 When you don't wash it, do you air it out?
- never or rarely
- occasionally
- regularly

18) Do you use a detergent:
- industrial?
- with ecolabel?
- homemade?
- I don't know?

19) Do you use:

- Fabric softeners? (yes / no/ I don't know)
- Stain removers? (yes/ no/ I don't know)
- Bleach? (yes/ no/ I don't know)

Housekeeping:

20) Do you sometimes mix several cleaning products at the same time to clean your interior?
- never or rarely
- occasionally
- regularly
- I don't know

21) Do you use products containing bleach?
- Never
- once a month maximum
- once a week maximum
- more than once a week
- I don't know

22) On the packaging of cleaning products, read:

A. Precautions for use?
- never or rarely
- occasionally
- regularly

B. Pictograms?
- never or rarely
- occasionally
- regularly

23) Do you remove dust with wet cleaning (cloth soaked in water)?
- never or rarely
- occasionally
- regularly

Furniture and decoration:

24) In the last twelve months, have you acquired/purchased:
- new cloth armchairs or sofas?
- new treated wood furniture?
- Second-hand furniture?

25) When you buy new furniture, do you air:
- more often than usual when you smell?
- more often than usual even when there is no smell?
- as often as usual?
- less than usual?

Do-it yourself and construction:

26) If, for example, you were to repaint a child's room in your home yourself, what attitude would you adopt/adopt?
- I look at the composition of the paintings
- I look at the pictograms of the paintings
- I am wearing a mask
- I keep pregnant women away from this room.

- I forbid the access of this room to the children
- I air this room more often than usual during the work.
- I air this room more often than usual for several weeks after the work is completed.
- I aerate my house more often than usual during the work.

Table S1. Practice on air renewal according to socio-demographic characteristics (n=554).

	Home ventilation in winter per week*			CMV cleaned at least once a year**		Housing temperature above 22 degrees**	
	1 to 3 times	4 or more times					
	OR [99 % CI]	OR [99 % CI]	p-value	OR [99 % CI]	p-value	OR [99 % CI]	p-value
**Age**	1.02 [0.98-1.07]	1.04 [1.00-1.08]	0.028	1.00 [0.97-1.04]	0.858	0.92 [0.85-0.99]	**0.004**
**Sex**							
men	1	1	0.408	1	0.083	1	0.633
women	0.97 [0.47-2.00]	1.6 [0.54-4.73]		1.62 [0.78-3.36]		0.81 [0.24-2.69]	
**Degree of urbanization of their residence**							
Rural area : < 2,000 people	1	1	0.892	1	0.253	1	0.452
2000 to 9999 people	1.04 [0.35-4.24]	0.8 [0.30-2.09]		1.57 [0.81-3.02]		0.66 [0.16-2.74]	
10000 to 49999 people	1.14 [0.32-4.11]	1.08 [0.14-8.48]		1.22 [0.60-2.49]		0.39 [0.04-4.12]	
≥50,000 people : Rennes	0.93 [0.3-2.91]	1.00 [0.44-2.27]		1.52 [0.8-2.88]		1.16 [0.34-3.93]	
**Living in a couple**							
no	1	1	0.369	1	0.447	1	0.108
yes	1.48 [0.71-3.11]	1.14 [0.63-2.05]		1.2 [0.64-2.23]		0.50 [0.16-1.56]	
**Education**							
Less than a French secondary school diploma	1	1	0.129	1	**<0.001**	1	0.011
French secondary school diploma	0.95 [0.32-2.82]	0.59 [0.24-1.49]		0.49 [0.22-1.11]		0.09 [0.01-0.72]	
Two or three-year university level	0.64 [0.24-1.68]	0.67 [0.24-1.83]		0.68 [0.28-1.66]		0.85 [0.22-3.25]	
Master's level or more	0.10 [0.36-2.76]	0.49 [0.14-1.73]		0.20 [0.10-0.39]		0.30 [0.05-1.62]	
**Having one or more children**							
no	1	1	0.069	1	0.094	1	0.169
yes	1.59 [0.91-2.78]	1.14 [0.66-1.96]		1.43 [0.82-2.49]		0.53 [0.16-1.79]	

**Expecting a baby**							
no	1	1	0.779	1	0.085	1	0.814
yes	0.71 [0.21-2.51]	0.76 [0.19-3.13]		2.29 [0.65-8.06]		1.16 [0.21-6.36]	

*Reference: less than once a week, **Reference: no.

Table S2. Practice on combustion appliances according to socio-demographic characteristics (n=554).

	Allowed indoor smoking*		Checked heating systems at least once a year*	
	OR [99 % CI]	p-value	OR [99 % CI]	p-value
**Age**	0.95 [0.89-1.02]	0.071	1.06 [1-1.12]	0.016
**Sex**				
men	1	0.628	1	0.206
women	0.88 [0.42-1.82]		1.32 [0.74-2.36]	
**Degree of urbanization of their residence**				
Rural area : < 2,000 people	1	0.480	1	0.015
2000 to 9999 people	1.46 [0.44-4.84]		1.71 [0.71-4.12]	
10000 to 49999 people	0.66 [0.1-4.4]		3.01 [1.25-7.26]	
≥50,000 people : Rennes	1.61 [0.49-5.33]		1.65 [0.68-4.00]	
**Living in a couple**				
no	1	**<0.001**	1	**0.006**
yes	0.26 [0.12-0.56]		1.75 [1.05-2.94]	
**Education**				
Less than a French secondary school diploma	1	0.162	1	0.100
French secondary school diploma	0.48 [0.15-1.55]		0.84 [0.4-1.78]	
Two or three-year university level	1 [0.32-3.15]		0.45 [0.16-1.25]	
Master’s level or more	0.61 [0.17-2.23]		0.92 [0.38-2.26]	
**Having one or more children**				

no	1	**<0.001**	1	**<0.001**
yes	0.25 [0.1-0.67]		2.9 [1.65-5.09]	
**Expecting a baby**				
no	1	0.405	1	0.119
yes	0.64 [0.16-2.64]		1.9 [0.64-5.59]	

*Reference: no.

Table S3. Hygiene products and cosmetics use according to socio-demographic characteristics (n=554).

	Check the composition of hygiene products*		Use an application to choose hygiene or cosmetic products*		Use five or more products daily*	
	OR [99% CI]	p-value	OR [99% CI]	p-value	OR [99% CI]	p-value
**Age**	1.00 [0.95-1.04]	0.79	0.98 [0.93-1.04]	0.38	0.96 [0.92-1.00]	0.012
**Sex**						
men	1	0.039	1	0.293	1	**<0.001**
women	1.85 [0.85-4.01]		1.57 [0.50-4.90]		8.30 [3.75-18.34]	
**Degree of urbanization of their residence**						
Rural area : < 2,000 people	1	0.187	1	0.241	1	0.579
2000 to 9999 people	0.72 [0.34-1.53]		0.33 [0.05-2.07]		0.68 [0.33-1.40]	
10000 to 49999 people	0.77 [0.38-1.56]		0.22 [0.01-5.33]		0.90 [0.47-1.75]	
≥50,000 people : Rennes	1.21 [0.61-2.40]		0.56 [0.21-1.49]		0.90 [0.52-1.56]	
**Living in a couple**						
no	1	0.838	1	0.323	1	0.055
yes	0.95 [0.48-1.87]		1.32 [0.63-2.80]		0.68 [0.40-1.15]	
**Education**						
Less than a French secondary school diploma	1	0.016	1	**0.002**	1	0.029
French secondary school diploma	0.95 [0.43-2.12]		1.61 [0.52-4.96]		1.75 [0.74-4.14]	

Two or three-year university level	1.79 [0.80-4.03]		1.82 [0.44-7.46]		1.36 [0.65-2.82]	
Master's level or more	2.67 [0.94-7.54]		3.71 [1.56-8.82]		0.71 [0.34-1.47]	
**Having one or more children**						
no	1	0.234	1	0.548	1	0.06
yes	0.80 [0.49-1.32]		1.18 [0.57-2.43]		0.66 [0.36-1.18]	
**Expecting a baby**						
no	1	0.884	1	0.586	1	0.994
yes	1.06 [0.35-3.20]		0.78 [0.23-2.66]		1.00 [0.46-2.18]	

*Reference: no.

Table S4. Pratices on textile according to socio-demographic characteristics (n=554).

	Wash textiles before first use*		Wash textiles with homemade or Eco-label laundry*	
	OR [99 % CI]	p-value	OR [99 % CI]	p-value
**Age**	1.03 [0.99-1.07]	0.038	1.03 [0.98-1.08]	0.156
**Sex**				
men	1	0.019	1	0.439
women	1.50 [0.96-2.34]		0.83 [0.43-1.59]	
**Degree of urbanization of their residence**				
Rural area : < 2,000 people	1	0.131	1	0.298
2000 to 9999 people	0.74 [0.33-1.68]		0.96 [0.49-1.89]	
10000 to 49999 people	0.68 [0.22-2.13]		0.66 [0.35-1.26]	
≥50,000 people : Rennes	0.51 [0.24-1.10]		0.82 [0.37-1.79]	
**Living in a couple**				
no	1	**0.008**	1	0.196
yes	1.66 [1.01-2.71]		1.43 [0.69-2.97]	
**Education**				
Less than a French secondary school diploma	1	0.210	1	**< 0.001**

French secondary school diploma	1.03 [0.54-1.98]		3.36 [1.56-7.26]	
Two or three-year university level	0.77 [0.33-1.81]		1.10 [0.35-3.49]	
Master's level or more	0.64 [0.33-1.26]		5.57 [1.47-21.12]	
**Having one or more children**				
no	1	0.072	1	0.437
yes	1.68 [0.79-3.56]		1.18 [0.68-2.05]	
**Expecting a baby**				
no	1	0.968	1	0.658
yes	0.98 [0.33-2.89]		0.83 [0.27-2.54]	

*Reference: no.

Table S5. Use and choice of cleaning products (n=554).

	Read precautions for use of cleaning products*			Mixed cleaning products*		
	occasionally	regularly		occasionally	regularly	
	OR [99% CI]	OR [99% CI]	p-value	OR [99% CI]	OR [99% CI]	p-value
**Age**	1.03 [0.99-1.07]	1.03 [0.99-1.07]	0.085	0.97 [0.9-1.03]	0.94 [0.89-0.98]	**0.003**
**Sex**						
men	1	1	0.030	1	1	0.879
women	0.93 [0.51-1.71]	1.98 [0.76-5.15]		1.06 [0.4-2.86]	1.24 [0.36-4.25]	
**Degree of urbanization of their residence**						
Rural area : < 2,000 people	1	1	**0.009**	1	1	0.540
2000 to 9999 people	0.96 [0.36-2.55]	0.68 [0.28-1.64]		1.07 [0.45-2.54]	0.93 [0.23-3.78]	
10000 to 49999 people	1.56 [0.43-5.75]	0.74 [0.09-6.49]		1.26 [0.32-4.9]	0.75 [0.15-3.65]	
≥50,000 people : Rennes	0.45 [0.17-1.17]	0.80 [0.40-1.59]		2.13 [0.72-6.30]	1.15 [0.38-3.55]	
**Living in a couple**						
no	1	1	0.585	1	1	**<0.001**

yes	1.19 [0.56-2.55]	0.91 [0.41-1.99]		0.50 [0.23-1.10]	0.40 [0.16-1.01]	
**Education**						
Less than a French secondary school diploma	1	1	0.239	1	1	0.105
French secondary school diploma	0.83 [0.40-1.72]	1.00 [0.55-1.81]		1.14 [0.44-2.97]	0.29 [0.07-1.17]	
Two or three-year university level	0.70 [0.38-1.27]	1.02 [0.33-3.18]		1.50 [0.40-5.69]	0.74 [0.24-2.24]	
Master's level or more	0.97 [0.39-2.44]	0.85 [0.24-2.94]		0.63 [0.22-1.87]	0.17 [0.02-1.26]	
**Having one or more children**						
no	1	1	0.638	1	1	0.342
yes	1.15 [0.67-2.00]	1.23 [0.66-2.28]		0.68 [0.34-1.36]	1.14 [0.40-3.28]	
**Expecting a baby**						
no	1	1	0.673	1	1	0.518
yes	1.23 [0.66-2.28]	0.73 [0.22-2.42]		0.72 [0.25-2.09]	0.46 [0.06-3.44]	

*Reference: Never or rarely.

Table S6. Attitude during renovation in a children room according to socio-demographic characteristics (n=554).

	Aerate more often during/after work*		Look at the composition of the products used for work*		Keep pregnant women away from the work room*		Prohibit access to the work room to children*	
	OR [99% CI]	p-value	OR [99% CI]	p-value	OR [99% CI]	p-value	OR [99% CI]	p-value
**Age**	0.96 [0.85-1.07]	**<0.001**	0.96 [0.93-0.997]	**0.002**	0.94 [0.90-0.97]	**<0.001**	0.96 [0.92-0.99]	**<0.001**
**Sex**								
men	1	0.376	1	0.191	1	0.493	1	0.139
women	2.03 [0.24-16.94]		1.44 [0.69-3.04]		0.86 [0.48-1.54]		1.52 [0.72-3.23]	
**Degree of urbanization of their residence**								
Rural area : < 2,000 people	1	0.420	1	0.940	1	0.673	1	0.981
2000 to 9999 people	0.15 [0.01-3.85]		0.96 [0.44-2.10]		1.02 [0.42-2.48]		0.88 [0.34-2.27]	
10000 to 49999 people	0.33 [0.01-20.46]		0.85 [0.37-1.94]		1.46 [0.50-4.28]		0.97 [0.32-2.98]	
≥50,000 people : Rennes	0.16 [0.01-3.55]		0.89 [0.38-2.07]		0.92 [0.37-2.25]		0.93 [0.38-2.27]	

**Living in a couple**								
no	1	0.098	1	0.716	1	0.029	1	0.056
yes	3.66 [0.47-28.79]		1.08 [0.61-1.91]		0.62 [0.35-1.10]		0.58 [0.28-1.22]	
**Education**								
Less than a French secondary school diploma	1	0.277	1	0.249	1	0.793	1	0.405
French secondary school diploma	0.59 [0.06-6.39]		0.94 [0.41-2.14]		1.18 [0.61-2.27]		1.12 [0.37-3.39]	
Two or three-year university level	5.97 [0.24-149.7]		1.57 [0.59-4.18]		1.07 [0.54-2.09]		0.91 [0.32-2.58]	
Master's level or more	1.46 [0.11-19.75]		1.35 [0.39-4.66]		0.93 [0.44-1.96]		0.63 [0.25-1.60]	
**Having one or more children**								
no	1	0.553	1	0.086	1	**0.001**	1	0.201
yes	1.61 [0.19-13.51]		0.59 [0.27-1.32]		0.4 [0.21-0.78]		0.70 [0.34-1.46]	
**Expecting a baby**								
no	1	0.451	1	0.461	1	0.601	1	0.249
yes	0.49 [0.04-5.96]		1.38 [0.43-4.41]		1.18 [0.52-2.68]		0.60 [0.19-1.93]	

*Reference: no.

Table S7.: Practice on indoor air according to perceptions (n=554).

	Home ventilation in winter per week*			CMV cleaned at least once a year**		Housing temperature above 22 degrees**		Allowed indoor smoking**		Checked heating systems at least once a year**	
	1 to 3 times	4 or more times									
	OR [99 % CI]	OR [99 % CI]	p-value	OR [99 % CI]	p-value	OR [99 % CI]	p-value	OR [99 % CI]	p-value	OR [99 % CI]	p-value
Have ever heard of indoor pollution											
No	1	1	0.079	1	0.739	1	0.617	1	0.784	1	0.064
Yes	1.04 [0.62-1.75]	1.64 [0.89-3.03]		0.90 [0.40-2.03]		0.78 [0.21-2.94]		1.09 [0.47-2.57]		1.44 [0.86-2.40]	
Perceived level of health risk of indoor chemicals											
≤4	1	1	0.532	1	0.605	1	0.058	1	0.382	1	0.309
≥5	1.45 [0.59-3.57]	1.40 [0.55-3.55]		1.21 [0.46-3.19]		0.44 [0.14-1.37]		0.70 [0.24-2.08]		0.75 [0.36-1.58]	

Ubiquity of environmental chemicals											
Disagree/mostly disagree	1	1	0.335	1	0.651	1	0.866	1	0.371	1	0.321
Agree	0.55 [0.25-1.18]	0.55 [0.19-1.6]		1.27 [0.63-2.53]		1.38 [0.27-6.97]		1.81 [0.50-6.57]		1.11 [0.55-2.24]	
Completely agree	0.67 [0.22-2.07]	0.53 [0.20-1.40]		1.18 [0.53-2.62]		1.19 [0.20-7.03]		1.38 [0.35-5.42]		1.43 [0.72-2.86]	
Perceived health risk for French of indoor pollution											
Low or medium	1	1	0.312	1	0.311	1	0.996	1	0.907	1	0.605
High or very high	1.37 [0.72-2.61]	1.34 [0.71-2.56]		1.22 [0.72-2.06]		1.00 [0.29-3.45]		0.96 [0.42-2.23]		1.14 [0.59-2.18]	

*Reference: less than once a week, **Reference: no.

© 2019 by the authors. Licensee MDPI, Basel, Switzerland. This article is an open access article distributed under the terms and conditions of the Creative Commons Attribution (CC BY) license (http://creativecommons.org/licenses/by/4.0/).
